# The composition of the gut microbiota is altered in biliary atresia with cholangitis

**DOI:** 10.3389/fsurg.2022.990101

**Published:** 2022-09-20

**Authors:** Lulu Zheng, Yibo Wu, Zhenhua Gong, Zhibao Lv, Weijue Xu, Qingfeng Sheng, Xiong Huang, Jiangbin Liu

**Affiliations:** Department of General Surgery, Shanghai Children’s Hospital, School of Medicine, Shanghai Jiao Tong University, Shanghai, China

**Keywords:** biliary atresia, composition, gut microbiota, Kasai operation, cholangitis

## Abstract

**Aim:**

To detect the composition of the gut microbiota in biliary atresia after Kasai surgery.

**Methods:**

Infants within six months after the Kasai operation who were diagnosed by cholangiography at Shanghai Children’s Hospital were enrolled in the study. Fecal samples were collected from diapers, placed into sterile tubes in the inpatient department or outpatient department and frozen at −80°C within half an hour. The gut microbiota was detected by 16S rRNA sequences. Then, the patients that were followed up to one year after the Kasai operation who suffered from cholangitis at least one time were grouped into the BAcho group, and the others were grouped into the BAnoncho group.

**Results:**

Nine of 18 BA patients were grouped into the BAcho group, and the others were grouped into the BAnoncho group. In the BAcho group, AST, ALT and GGT were significantly increased compared to the BAnoncho group. The number of total OTUs (operational taxonomic units) in feces was more elevated in the BAnoncho group than in the BAcho group. In the BAnoncho group, the Chao index at the OTU level was significantly increased compared to that in the BAcho group (66.37 ± 21.5 vs. 45.64 ± 11.25, *p* = 0.02 < 0.05). *Bifidobacterium* was the most abundant genus in the BAnoncho group, accounting for 22.14%, and *Klebsiella* accounted for 22.74% in the BAcho group. Compared with the BAnoncho group, *Bacteroides* was significantly decreased in the BAcho group (*p* = 0.037).

**Conclusion:**

The composition of the gut microbiota was different between BA with cholangitis and BA without cholangitis.

## Introduction

Biliary atresia (BA) is a progressive bile duct sclerosis disease with cholestasis that results in liver fibrosis (1). The incidence of BA ranges from 0.5–5:10,000 worldwide. The pathogenesis of BA, such as viral infection, gene mutation, autoimmunity, and abnormalities in bile duct development, is unclear (2). Kasai surgery, which was first performed by Kasai in Japan, is one of the main treatments for BA. However, approximately 70%–80% of children with liver cirrhosis after successful surgery still need liver transplantation (3–6). Reflux cholangitis, which is caused by the gut microbiota, is one of the risk factors for liver fibrosis after Kasai surgery. In addition, more than 60% of patients suffer from cholangitis within the first year after Kasai surgery (7, 8). Therefore, the purpose of the study was to investigate the distribution of the gut microbiota in cholangitis within the first year after Kasai surgery.

## Methods

### Patient samples

Type III biliary atresia infants from Shanghai Children’s Hospital were enrolled in the study. The biliary atresia was diagnosed by intraoperative cholangiography. After Kasai operation, cefoperazone sulbactam and metronidazole were given intravenously for 4 weeks, and then cefixime or sulfamethoxazol were taken orally in turn 5 mouths (change of oral antibiotic every week). All BA patients who were within 6 months after Kasai surgery with oral cefixime or cotrimoxazole were recruited to determine the gut microbiota between 2019.06 and 2020.06. All patients were followed up to one year after hepatic portoenterostomy. The exclusion criteria included intravenous antibiotics, oral probiotics and the loss of follow-up infants. The BA infants who suffered from at least one episode of cholangitis in the follow-up period were grouped as BAcho (BA patients with cholangitis), and the others were grouped as BAnoncho (BA patients without cholangitis). Meanwhile, the general characteristic information of all patients was collected. The definition of cholangitis is at least two of the following three points: (a) fever (temperature ≥38.5°C) with no other reasons, (b) jaundice or stool with lighter color, and (c) blood testing with elevated leukocytosis or C-reactive protein.

### Fecal sample collection and microbiota extraction

Fecal samples were collected from the diaper, placed into sterile tubes in the inpatient department or outpatient department and frozen at −80°C within half an hour. All samples were obtained from Shanghai Children’s Hospital in China. This study was approved by the ethics committee of the hospital. Genomic DNA extraction, PCR amplification, library preparation, and Illumina sequencing were performed according to methods described previously (9). In brief, total microbial DNA was extracted using a QIAamp DNA stool minikit (Qiagen, Germany). The extracted genomic DNA was PCR (polymerase chain reaction) amplified with barcoded primers (forward primer, 5′-ACT CCT ACG GGA GGC AGC AG-3′; reverse primer, 5′-GGA CTA CHV GGG TWT CTA AT-3′) targeting the 16S rRNA V3–V4 region. Water samples that had undergone the same procedures of DNA extraction and PCR amplification were used as a control. An equal amount of DNA from each sample was pooled and verified using an Agilent 2100 bioanalyzer (Agilent, USA). The data were analyzed on the free online Majorbio Cloud Platform (www.majorbio.com).

### Statistical analysis

Differences in means were tested by the independent Student’s t test or the Wilcoxon rank-sum test between the two groups using SPSS 22. A *p* value of <0.05 was considered statistically significant. Bar graphs and photographs were processed by GraphPad Prism 8.

## Results

### Patient and clinical characteristics

Eighteen BA patients were recruited for our study. Nine infants were grouped into BAcho, and the others were grouped into BAnoncho. Demographic data on clinical features are shown in Table 1. In the BAcho group, aspartate transferase, alanine transferase, and gamma-glutamyl transpeptidase levels in blood were significantly increased compared to those in the BAnoncho group. There were no differences between the two groups in terms of surgery age, collection time, direct bilirubin, total bilirubin, alkaline phosphatase or total bile acid. In the BAcho group, the yield of blood culture was 0. Five of nine patients in the BAcho group suffered from more than two cholangitis episodes within one year. Biochemical figures between BAcho group and BAnoncho group one mouth after Kasai surgery were also shown in Supplementary Table S2.

**Table 1:** Biochemical figures between BAcho group and BAnoncho group.

			BAcho				BAnoncho		
	*N*	MIN	MAX	M (SD)	*N*	MIN	MAX	M (SD)	*p*
Age (day)	9	51	91	63.11 (14.92)	9	31	71	58.44 (13.58)	0.498
CT (day)	9	30	146	81.33 (35.27)	9	30	180	98.67 (48.99)	0.402
DB (uM)	9	6.3	93.2	58.39 (33.11)	9	1.7	110.2	28.23 (45.27)	0.126
TB (uM)	9	14.5	157.39	101.01 (59.18)	9	6.6	205.34	53.99 (83.66)	0.188
AST (U/L)	9	132	317	226 (95.7)	9	47	274	98.33 (76.7)	0.007*
ALT (U/L)	9	123	485	297.11 (125.38)	9	31	292	101.78 (81.32)	0.001*
GGT (U/L)	9	146	1893	1043.56 (513.27)	9	70	769	330.11 (248.16)	0.002*
ALP (U/L)	8	225	1220	509.25 (338.18)	9	224	631	425 (163.82)	0.515
TBA	7	64	326	143.57 (91.15)	8	12	226	97.88 (88.6)	0.343

BAcho, biliary atresia with cholangitis group; BAnoncho, biliary atresia without cholangitis group; AGE, surgery time; CT, collectting stool time; DB, direct bilirubin; TB, total bilirubin; AST, aspartate transferase; ALT, alanine transferase; GGT, gamma-glutamyl transpepetidase; ALP, alkaline phosphatase; TBA, total bile acid.

* *p* < 0.01.

### Gut microbiota distribution in BA

**Figure 1:**
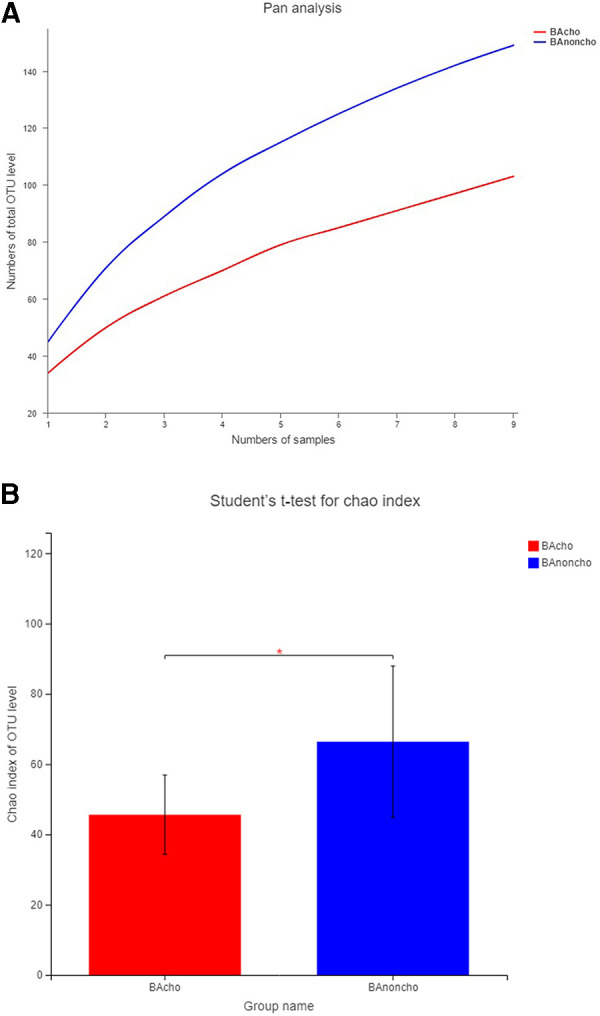
The OUT level in BAcho and BAnoncho. (**A**) The numbers of total OTU level were increasing with the numbers of samples. The numbers of OTU level in BAnoncho were more than those in BAcho in same numbers of samples. (**B**) In BAnoncho group, Chao index of OTU level was significantly increased compared to the BAcho group. (*p < 0.05, OTU: Operational Taxonomic Unit, defined by >97% 16S rRNA sequence similarity. Chao index:using for calculating the Community richness).

**Figure 2:**
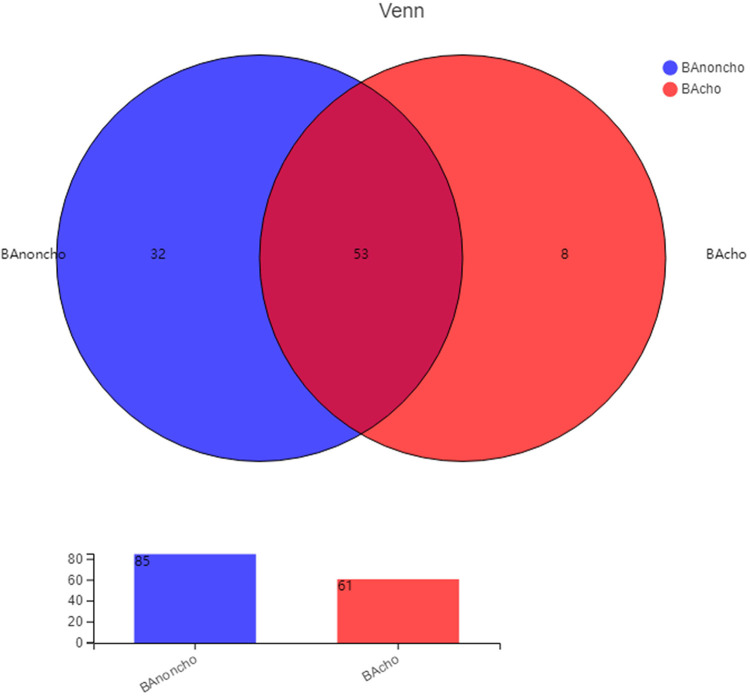
The kinds of gut microbiota in BAnoncho and BAcho group on genus level. There were 85 kinds of gut microbita in BAnoncho and 61 kinds in BAcho. There were 8 kinds of microbita on genus level only detected in BAcho group.

**Figure 3:**
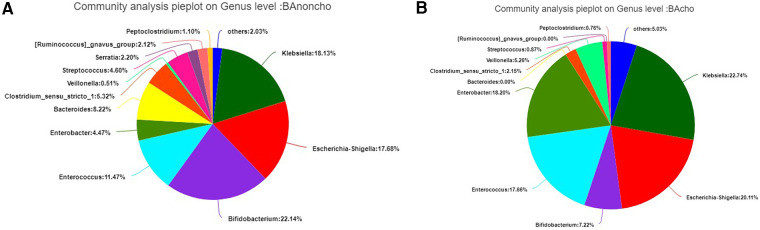
(**A**) The distribution of gut microbiota on genus level in BAnoncho. *Bifidobacterium* accounted for 22.14%. *Klebsiella* occupied 18.13%. *Escherichia-shigella* accounted for 17.68%. (**B**) The distribution of gut microbiota on genus level in BAcho. *Klebsiella* accounted for 22.74%. *Escherichia-Shigella* occupied 18.13%. *Enterococcus* accounted for 17.68%.

**Figure 4:**
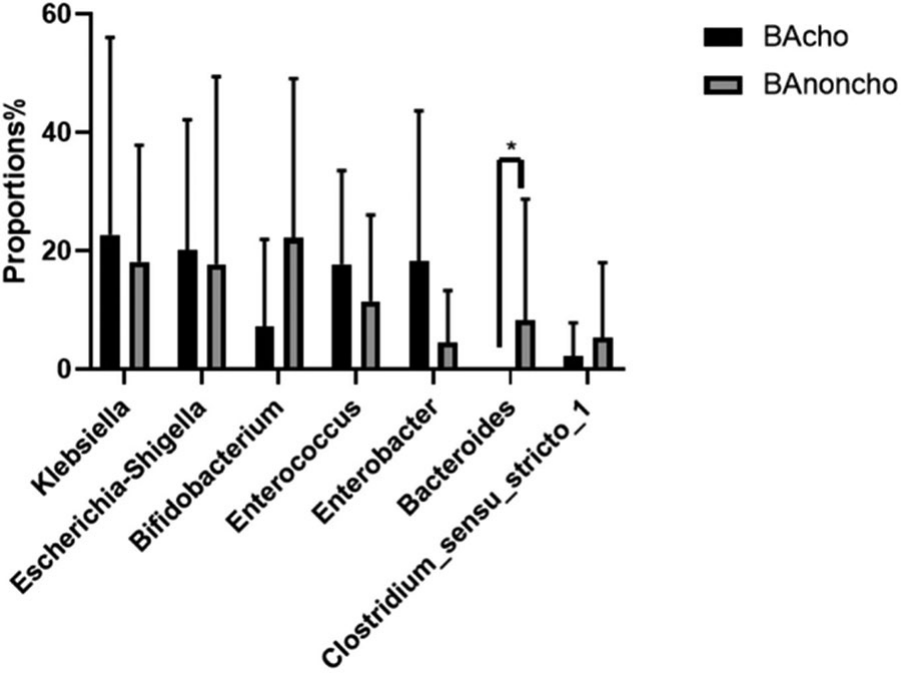
Wilcoxon rank-sum test bar plot on genus level between BAcho and BAnoncho. There was no difference on Klebsiella, Esherichia-Shigella, Bifidobacterium, Enterococcus, Enterobacter, Clostridium-*sensu*-*stricto*-1 between BAcho and BAnoncho group. Compare to BAcho, Bacterouides was significancely increased in BAnoncho. (*p < 0.05).

A total of 714,284 raw sequence reads were produced from 18 stool samples. The average number of reads per sample was 39682 ± 11062. The average length was 445 ± 4.9 bp (Supplementary Table S1). With the increasing number of samples, the number of total OTUs (operational taxonomic units) was more elevated in the BAnoncho group than in the BAcho group (Figure 1). In the BAnoncho group, the Chao index at the OTU level was significantly increased compared to that in the BAcho group (66.37 ± 21.5 vs. 45.64 ± 11.25, *p* = 0.02 < 0.05). There were 85 genera in the BAnoncho group and 61 in the BAcho group when less than 1% of genera were merged (Figure 2). In addition, 8 types of microbiota were detected at the genus level only in the BAcho group (shown in Supplementary Figure S1). The distribution of gut microbiota at the genus level in the BAcho and BAnoncho groups is shown in Figure 3. *Bifidobacterium* was the most abundant genus in the BAnoncho group, accounting for 22.14% (Figure 3). However, *Klebsiella,* which was the most abundant genus, occupied 22.74% in the BAcho group. Compared to the BAnoncho group, *Bacteroides* was significantly decreased in the BAcho group (0.0033 ± 0.0048 vs. 8.224 ± 20.48, *p* = 0.037). There was no difference in *Klebsiella, Escherichia-Shigella, Bifidobacterium, Enterococcus, Enterobacter, or Clostridium-sensu-stricto-1* between the two groups (Figure 4).

## Discussion

Cholangitis is the most common complication in BA after the Kasai operation. Katawaetee Decharun, MD (10) reported that the morbidity of cholangitis ranged from 15% to 62% with antibiotics. In most case series, nearly 90% of cholangitis patients experience episodes of cholangitis within the first year after the Kasai operation (11, 12). Qianfu Luo reported that serum bacteria such as *Escherichia coli*, *Klebsiella pneumoniae*, *Shigella fexneri*, and *Enterobacteriaceae* bacterium in cholangitis patients were similar to those in the gut (13). The main pathogenic mechanism of cholangitis may be bacterial translocation from the enteric tract, which may be caused by an insufficient volume of bile flow (14). In our study, there were 9 patients suffering from cholangitis, which accounted for 50%, and the incidence rate of cholangitis was similar to that in previous reports (15). Bacteria that cause cholangitis can be detected by blood culture, but the positivity rate is very low, ranging from 8.9% to 35.1% (16, 17). In our study, the yield of blood culture was zero. The main reason for the result is that the number of cases enrolled in the study was too small or because all of the patients were on oral antibiotics when the fecal samples were collected.

There are 100 trillion microorganisms of more than 1,000 types in the healthy human gut. The gastrointestinal microbial flora is affected by many factors, including diet, antibiotics, bile acid and delivery (18, 19). In turn, gut microbiota have a direct effect on human health by themselves and byproducts (18). Due to the diversity of affected factors, the gastrointestinal microbial flora in pediatric patients is very different from that in adults (20, 21). A study reported that in BA patients, the microbial diversity and gut primary and secondary bile acids were significantly reduced compared with those in healthy controls (22). It has been speculated that bile acid deficiency and abnormal bile acid metabolism may have altered the gut environment, leading to a shift in the gut microbiota composition (23). Yizhong Wang et al. reported that the diversity was significantly lower in infants with cholestatic jaundice than in healthy controls (9). In our study, the diversity of the fecal microbiota was significantly reduced in the BAcho group compared to the BAnoncho group. However, the blood total bile acid was similar between the BAcho group and BAnoncho group, and bile acid may not be the main factor affecting the diversity of the fecal microbiota in the BA after the Kasai operation.

To our knowledge, this is the first study to detect the composition of gut microbiota between the BAcho and BAnoncho groups by 16S rRNA sequencing. Zheng (22) reported that in BA, microbial dysbiosis was characterized by the enrichment of facultative anaerobes. Facultative anaerobes, such as *Streptococcus*, *Klebsiella* and *Enterococcus*, are considered potential pathogens that correlate with liver function indices in BA. In our study, *Klebsiella* was also increased in the two groups, which may be manipulated by antibiotics. The composition of *Bacteroides* accounted for 8.2% in the BAnoncho group compared with 0% in the BAcho group. The abundance of *Bacteroides* was very different in different alcohol feeding models, and it was found that the *Bacteroides* were relatively increased in intragastric feeding with alcohol, while *Bacteroides* were decreased in mice with chronic *ad libitum* ethanol feeding of the Lieber-DeCarli ethanol liquid (24–26). The abundance of *Bacteroides* was decreased in alcoholics. Ley considered that *Bacteroides* is decreased in human obesity (27). Due to the complexity of the fecal microbiota, the function of *Bacteroides* requires further study. We also found that *Bidfidobacterium,* which is considered a probiotic, had the highest proportion in the BAnoncho group. Tien-Hau Lien (28) reported that *Lactobacillus casei Rhamnosu,* a probiotic, was as effective as antibiotics in preventing cholangitis in BA. Recently, Ewa Orowska found (29) that the *Lactobacillus casei Rhamnosu* group had a lower rate of cholangitis after the Kasai operation than the placebo group, although the difference was not statistically significant in a randomized, double-blind, placebo-controlled trial. It is possible that probiotics for preventing cholangitis in BA with the Kasai operation may be a new therapy in the future.

## Conclusion

The composition of the gut microbiota was different between BA with cholangitis and BA without cholangitis.

## Data Availability

The datasets presented in this study can be found in online repositories. The names of the repository/repositories and accession number(s) can be found in the article/**Supplementary Material**.
