# Supplementary material for: The composition of the gut microbiota is altered in biliary atresia with cholangitis
Source: Front Surg. 2022 Sep 20;9:990101. doi: 10.3389/fsurg.2022.990101 (PMC9632985; doi:10.3389/fsurg.2022.990101)
Supplement: Supplementary file 2 [file Table2.docx]

|  |  |  | BAcho |  |  |  |  | BAnoncho |  |  |
| --- | --- | --- | --- | --- | --- | --- | --- | --- | --- | --- |
|  | N | MIN | MAX | M(SD) |  | N | MIN | MAX | M(SD) | P |
| DB(uM) | 9 | 31.8 | 118.1 | 84.71(44.37) |  | 9 | 12.6 | 106.4 | 49.31(45.27) | 0.081 |
| TB(uM) | 9 | 54.27 | 322.78 | 146.22(82.22) |  | 9 | 19.2 | 188.09 | 88.91(63.41) | 0.117 |
| AST(U/L) | 9 | 175 | 1115 | 331.11(104.17) |  | 9 | 68 | 239 | 145.56(84.03) | 0.001** |
| ALT(U/L) | 9 | 194 | 520 | 356.33(307.84) |  | 9 | 78 | 323 | 143.11(63.64) | 0.074 |
| GGT(U/L) | 9 | 135 | 1374 | 678.44(506.18) |  | 9 | 457 | 1450 | 854.22(349.76) | 0.404 |
| ALP(U/L) | 9 | 276 | 564 | 424.67(120.48) |  | 9 | 224 | 851 | 408.44(181.69) | 0.826 |
| TBA | 5 | 63 | 431 | 201(141.5) |  | 5 | 54 | 226 | 149.8(62.26) | 0.48 |
