# Supplementary figures and images for: The composition of the gut microbiota is altered in biliary atresia with cholangitis
Source: Front Surg. 2022 Sep 20;9:990101. doi: 10.3389/fsurg.2022.990101 (PMC9632985; doi:10.3389/fsurg.2022.990101)

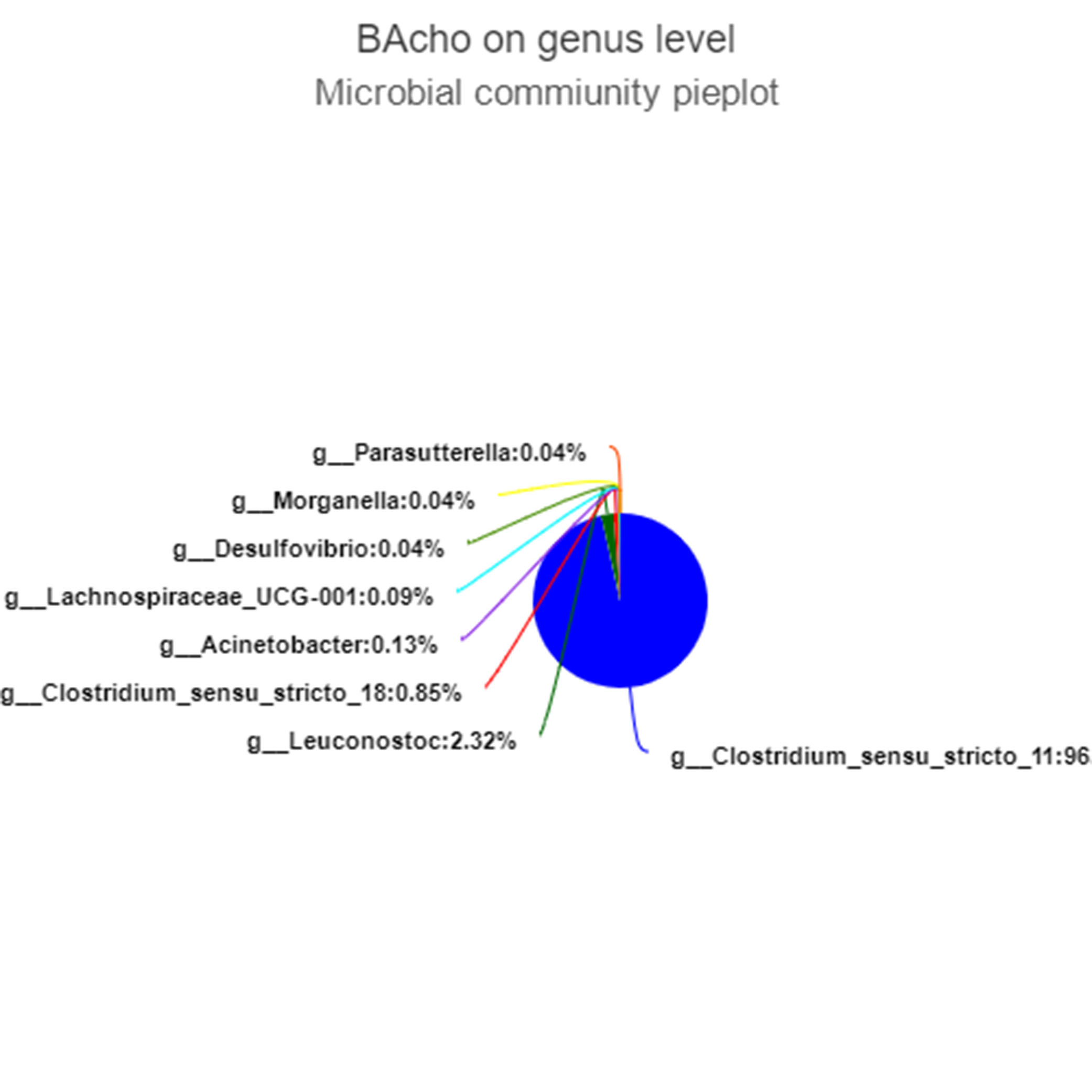

Supplement: Supplementary file 3 [file Image1.jpeg]
